# Large Format Histology May Aid in the Detection of Unsuspected Pathologic Findings of Potential Clinical Significance: A Prospective Multiyear Single Institution Study

**DOI:** 10.1155/2012/532547

**Published:** 2012-09-20

**Authors:** Matthew R. Foster, Lauren Harris, Karl W. Biesemier

**Affiliations:** Department of Pathology, Centra Health and Pathology Consultants of Central Virginia, 1914 Thompson Drive, Lynchburg, VA 24501, USA

## Abstract

Large format histology offers several unique advantages over traditional tissue processing. Over 12 years of experience with this technique provide insight into its limitations and benefits. We conducted a prospective multiyear analysis of the potential advantages of large format histology. 656 cases were examined prospectively over an eight-year period. In 172 cases the sign-out pathologist documented an unexpected finding of potential clinical significance as present only on the large format sections and not present on the accompanying standard format slides. These include closer margins, a change in size or extent of disease, and previously undocumented invasive and/or in situ carcinoma. Based on over a decade of experience and eight years of data, our results demonstrate that a quarter of cases had an unexpected finding of potential clinical significance that may not have been fully realized without the use of the large format technique.

## 1. Background

Large format histology (LFH), a form of tissue processing utilizing large paraffin blocks, a large format microtome, and a large glass slide accommodate a large contiguous portion of breast tissue. The methods and history of the large format technique have been documented elsewhere [1–3]. Our laboratory has utilized this technique for over 12 years and have realized an advantage afforded by the large format process of examination of a larger continuous intact portion of breast tissue. In our laboratory, the typical large format tissue sample measures up to 6.0 × 8.0 × 0.5 cm with a glass slide measuring 12.0 × 8.5 cm as compared to a standard tissue size of 2.0 × 2.5 × 0.3 cm and standard slide measurement of 2.5 × 7.5 cm. Most previously recognized benefits result from the obvious size difference. As reported elsewhere large format histology facilitates examination of large, diffuse, or multifocal processes in addition to facilitating evaluation of the adequacy of excision, locating and quantifying residual disease in the neoadjuvant setting, and enhancing pathologic-imaging correlation [4, 5]. Our ongoing experience continues to prove that the large format technique can be successfully incorporated into a community-based practice without significant increases in cost, staffing, or time.

 However, to our knowledge, no prospective data exist about the potential diagnostic and therapeutic benefits of large format histology versus standard format tissue processing in a community hospital setting. In an attempt to quantify what observable differences or advantages may be realized both pathologically and clinically from the large format technique, we designed a prospective multiyear analysis of large format histology compared with standard format histology.

## 2. Methods

 From January 2004 to May 2012, we conducted a prospective analysis of the potential benefit of large format histology. Using an assessment sheet provided to the sign-out pathologist at the time of the original evaluation, we compared large format histology to standard format histology. We relied on the sign-out pathologist to record the findings on a standardized data collection sheet, in particular asking them to note any significant findings which were not anticipated preoperatively and were not present on the accompanying standard format slides. We did not provide a definition of a significant finding during the study and instead relied on the pathologist's judgement to note specific findings as appropriate in order to allow for a more broad scope of what might be considered significant. However, in evaluating the data at the conclusion of the study we defined a significant finding as one which had the potential to alter clinical management. A total of seven pathologists all in the same community practice, including two of the authors of this paper (M. R. Foster and K. W. Biesemier), participated in the study. Six of the seven pathologists participated for the entire eight-year duration of this study. Review of the submitted data sheets revealed commonly reported unexpected findings. These include unanticipated invasive carcinoma or ductal carcinoma in situ (DCIS), an unanticipated change in size or extent of the lesion when compared to the preoperative clinical and/or radiographic impression, and an unanticipated closer surgical margin.

 The number of large format blocks varied from case to case, dependent on the size of the lesion, the diagnosis (DCIS versus invasive), and the nature of the specimen (e.g., a reexcision with a visible biopsy cavity or a specimen resected after neoadjuvant therapy). The selection of which portion of the specimen, if any, submitted for LFH was made at the time of gross examination by the sign-out pathologist and assistant (PA), if applicable. In general these ranged from one to three blocks. Traditional small blocks were submitted on most cases for directed sampling, comparison with the large format blocks, and for potential supplemental prognostic/predictive biomarker studies (ER, PR, Her2-neu, Oncotype DX, etc.). When the lesion of interest was included only in the large format blocks, conventional small slides from directed areas were prepared from the large format blocks for special studies. Rare cases, typically small lumpectomies with a reported large region of DCIS which was not completely identified on gross examination, may have been entirely submitted for large format histology. This pathologist directed balanced approach to the examination of breast specimens utilizing a combination of LFH and conventional small block histology that has been enthusiastically endorsed by our laboratory for over a decade, providing enhanced pathologic mammographic correlation as previously described [4].

## 3. Results

**Figure 1:**
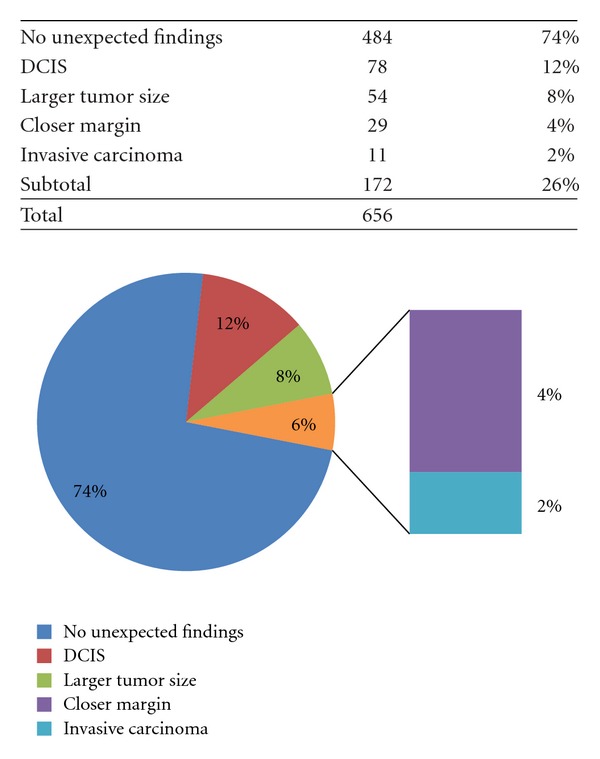
Distribution of potentially clinically significant unexpected findings in 656 large format cases from 2004 to May 2012. Tumor is defined as DCIS or invasive.

From 2004 to 2012 a total of 656 large format cases were analyzed prospectively. Both standard format and large format slides were utilized in most cases. In 593/656 (90%) the original sign-out pathologist felt large format histology was helpful in establishing the pathologic diagnosis and allowing for accurate assessment of currently accepted parameters for the examination of specimens from in situ and invasive breast cancer as outlined in the CAP guidelines for examining breast carcinoma [6]. In 172/656 (26%) of cases an unexpected finding was present on the large format slides which was not seen on the accompanying standard format slides from the same case. As reflected in Figure 1, these include 78 (12%) with unexpected DCIS, 54 (8%) with unexpected change in size or extent of the DCIS or invasive carcinoma, 29 (4%) with closer margin than expected based on initial examination and imaging, and 11 (2%) with unexpected invasive disease. Unexpected invasive disease was generally microinvasion associated with DCIS or very small tumor foci not previously detected by imaging.

## 4. Discussion 

One obvious limitation of this study remains how many of the unexpected findings would have been identified on standard format sections without the use of large format slides. It certainly remains plausible that some if not all observed findings may have been identified if a similar specimen area were submitted via standard format sections. However, others have shown this to be an inefficient means of examining breast tissue [7, 8]. Our experience is similar. We continue to believe that the use of large format histology allows for a timely and efficient means to assess a broad contiguous region of breast tissue. Further, as documented elsewhere, the large format technique can allow for better correlation with imaging studies [4, 9]. Our data collection forms did not specify the degree to which LFH findings may have altered TNM classification or specific histologic features of any newly discovered invasive disease. These details may be further evaluated by retrospective review of individual cases with significant findings.

 We did not exclude from analysis cases in which only large format sections were submitted. However, we estimate this to be a small subset, representing less than 10 total cases, and we believe inclusion of this small number does not significantly alter our results or conclusions.

 Our data show that the large format technique enabled the sign-out pathologist to identify potentially significant previously unsuspected findings and allowed for a more complete and accurate assessment of the extent to which in situ and invasive cancer impacts the de novo breast architecture. We continue to realize several unique advantages which large format histology offers over traditional tissue processing. Over 12 years of experience with large format histology provide insight into its limitations and benefits. Based on over a decade of experience and eight years of data, our results demonstrate that over a quarter of our cases had an unexpected finding that may have not been fully realized without the use of the large format technique.
